# Adjuvant trastuzumab duration trials in HER2 positive breast cancer – what results would be practice-changing? Persephone investigator questionnaire prior to primary endpoint results

**DOI:** 10.1186/s12885-018-4307-8

**Published:** 2018-04-05

**Authors:** Louise Hiller, Janet A. Dunn, Shrushma Loi, Anne-Laure Vallier, Donna L. Howe, David A. Cameron, David Miles, Andrew M. Wardley, Helena M. Earl

**Affiliations:** 1 Warwick Clinical Trials Unit, University of Warwick, Coventry, UK; 2 Addenbrooke’s Hospital, Clinical Trials Unit, Cambridge, UK; 3 University of Edinburgh, Edinburgh, UK; 4 Mount Vernon Cancer Centre, Northwood, UK; 5 The Christie Hospital NHS Foundation Trust and Faculty of Biology, Medicine and Health, University of Manchester, Manchester, UK; 6 Department of Oncology, University of Cambridge, Cambridge, UK; 7 NIHR Cambridge Biomedical Research Centre and Cambridge Breast Cancer Research Unit, Cambridge, UK

**Keywords:** Breast Cancer, HER2 positive, Adjuvant trastuzumab duration, Trastuzumab cardiotoxicity, Investigator questionnaire

## Abstract

**Background:**

Twelve months treatment is the current standard of care for adjuvant trastuzumab in patients with HER2 positive early breast cancer however the optimal duration is not known. Persephone is a non-inferiority randomised controlled trial comparing 6- to 12-months of trastuzumab. In this trial there will be a trade-off between a possible small decrease in disease-free survival (DFS) with 6-months and reduced cardiotoxicity and cost.

**Methods:**

A structured questionnaire asked clinicians who had recruited patients into the Persephone trial about their prior beliefs with regards to the clinical effectiveness of trastuzumab and cardiotoxicity profile, in the comparison of 6- and 12-month durations.

**Results:**

Fifty-one clinicians from 40 of the 152 Persephone sites completed the questionnaire. 30/50 responders (60%) believed that 6-months trastuzumab would give the same 4-year DFS rate as 12-months trastuzumab, with 21/50 (42%) holding this belief across all breast cancer subsets. In addition, 46/49 responders (94%) reported expecting to change their clinical practice to 6-months, with their prior beliefs (most commonly 85% 4-year DFS rate with 6-months) being greater than their lowest acceptable rate (most commonly 83% 4-year DFS rate with 6-months). Low levels of cardiotoxicity were expected with both 6 and 12-months trastuzumab, with the majority expecting lower levels with 6-months. With increasing hypothesised differences of cardiotoxicity rates between the two durations, significantly lower levels of 4-year DFS with 6-months trastuzumab were deemed acceptable (*p* < 0.0001).

**Conclusion:**

Most responders believe that 6-months trastuzumab is adequate, both overall and within each subset of breast cancer, and plan to change their clinical practice if the Persephone results support their prior belief. An individual patient meta-analysis of the duration trials would give greater precision to estimates of the differences in efficacy and toxicity, and adequate statistical power to establish a 2% level of non-inferiority for 6-months adjuvant trastuzumab.

**Electronic supplementary material:**

The online version of this article (10.1186/s12885-018-4307-8) contains supplementary material, which is available to authorized users.

## Background

Adjuvant trastuzumab treatment is proven to improve disease-free survival (DFS) and overall survival (OS) in HER2 positive early breast cancers. The results of the US and international adjuvant trastuzumab trials published in 2005–2011 [1–4] established 12 months of adjuvant trastuzumab as the clinical standard. In addition the HERA trial has recently confirmed that 24 months treatment does not further improve DFS and OS but does increase cardiac toxicity [5]. Investigation is still ongoing into shorter durations of adjuvant trastuzumab that could potentially provide an optimal balance between efficacy, cost and cardiac / other toxicity. Trials of trastuzumab duration comparing shorter durations to 12 months include PHARE [6], the HORG trial [7] and Persephone, which all compare 6 versus 12 months. In addition the Short-HER (NCT00629278) and SOLD (NCT00593697) trials have investigated 9 weeks versus 12 months duration. However, the PHARE trial, reported in 2013 [6], the HORG trial, reported in 2015 [7] and the Short-HER trial presented in 2017 [8], all failed to prove non-inferiority. The Persephone trial, completed target recruitment of 4000 patients in July 2015 and the primary endpoint results of DFS non-inferiority in an event-driven analysis are expected by June 2018.

The type, severity and length of increased risk of cardiotoxicity with trastuzumab treatment is also still being investigated with regard to the different treatment durations alongside various chemotherapy regimens. A number of trials have reported results by different categories: New York Heart Association Functional Classifications; absolute or relative declines in left ventricular ejection fraction (LVEF) percentages; signs and / or symptoms of congestive heart failure (CHF); new use or alteration of cardiac medication; and finally prevalence of cardiac deaths attributable to trastuzumab. The cardiac data from the HERA trial [5, 9–12] and the US studies [13, 14] show a clear relationship between the duration of trastuzumab exposure and incidence of cardiac dysfunction. This was confirmed both in PHARE [15] and the cardiac analysis of the first 2500 patients in the Persephone Trial [16].

There are plans for an international trastuzumab duration meta-analysis which will aim to further investigate the benefits of the different durations of trastuzumab treatment in breast cancer patients as a whole as well as in specific pre-specified subsets. The Persephone, PHARE and Short-HER Trials Groups have been in discussion with regards to this future meta-analysis, and a survey was proposed to establish clinicians’ prior beliefs with regard to the level of non-inferiority deemed acceptable in order to change their current prescribing practice.

To address this issue, a questionnaire was designed to ask clinicians who had recruited patients into Persephone about their current clinical beliefs of the effectiveness and cardiotoxicity profile of 6 and 12 months adjuvant trastuzumab treatment in the HER2 breast cancer setting. The aim was to inform not only the potential practice-changing impact of the Persephone trial, but also on the most appropriate non-inferiority limits to define for a future meta-analysis of the ‘12 month trastuzumab versus less’ trials for further investigation into pre-specified subsets of patients.

## Methods

Between May 2015 and July 2016, members of the Persephone trial team developed a structured questionnaire (see Additional file 1) to ask clinicians about their prior beliefs of the effectiveness and cardiotoxicity profile of adjuvant trastuzumab treatment in the HER2 positive early breast cancer setting, specifically with regards to the comparison between 12 month and 6 month duration.

The 20-point questionnaire comprised questions relating to five areas: (i) levels of DFS with the two treatment durations; (ii) the relative efficacy of 6 and 12 months trastuzumab in various specified subsets of patients; (iii) rates of cardiotoxicity with 6 and 12 months trastuzumab; (iv) acceptable trade-offs between decreases in cardiotoxicity rates and decreases in DFS; (v) any aspect of the comparison between various durations of trastuzumab treatment. Responders were allowed to remain anonymous, although all did include their names on the returned questionnaires.

### Statistical methods

Frequencies and percentages are used to display responses to individual questions, with bubble plots illustrating responses to linked questions. Box and Whisker plots are used to display respondents’ acceptable levels of 4-year DFS within the various scenarios, and random effects modelling was applied. Results were presented graphically as the mean respondent’s levels (and 95% confidence intervals (CI)) over the different scenarios, as predicted by the model.

## Results

### Respondents

In July 2016, the questionnaire was circulated to the 152 randomising hospitals for the Persephone trial. By December 2016, responses had been received from 51 clinicians at 40 of the hospitals.

### Perceptions of 4-year DFS

**Fig. 1:**
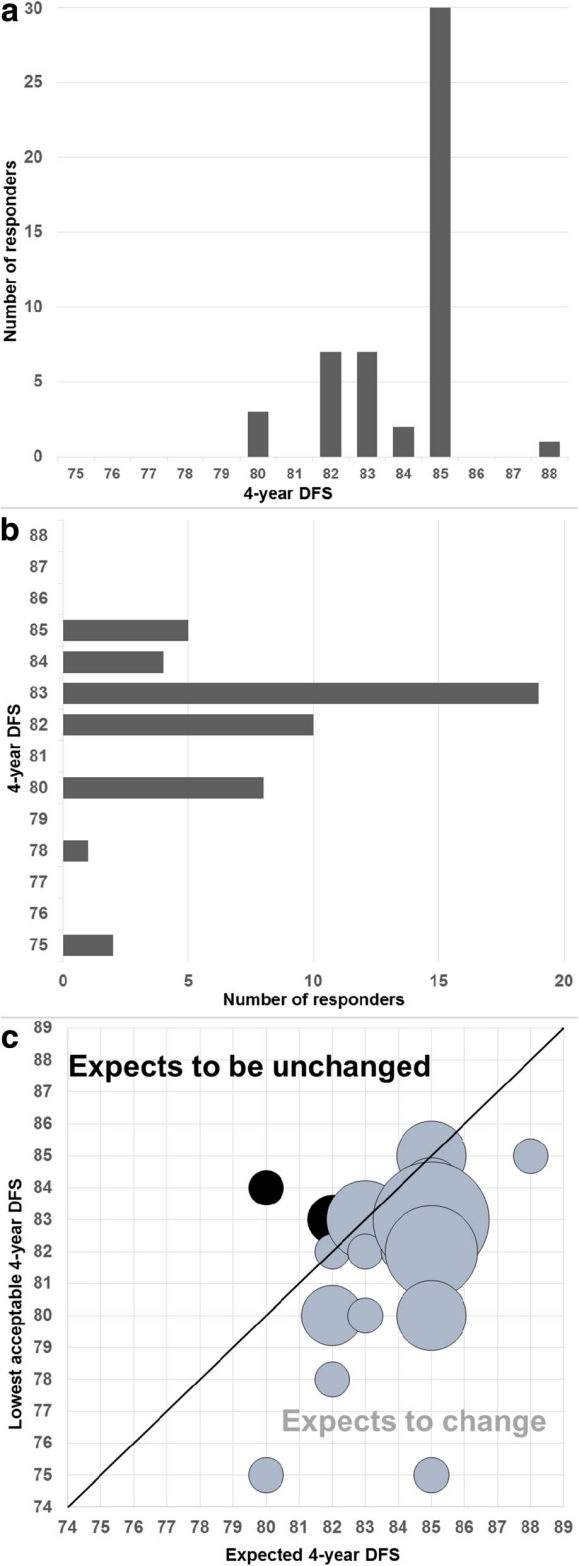
6-months trastuzumab: Opinions on 4-year DFS. **a** Expected 4-year DFS of patients receiving 6-months trastuzumab. **b** Lowest acceptable 4-year DFS to change practice to 6-months trastuzumab. **c** Expected vs lowest acceptable levels

When asked about their perceptions of 4-year DFS with 6-months trastuzumab, 60% of responders (30 of the 50 who responded to this question), believed it to be 85%, with responses ranging between 80 and 88% (Fig. 1a). When asked what the lowest level of 4-year DFS was that they would be comfortable with in order to change their current practice to 6-months trastuzumab, the most common response was 83% (19/49 responders (39%), range 75–85%, Fig. 1b). Only 3 responders (6% of the 49 who answered both questions) quoted expectations of 4-year DFS lower than their stated lowest acceptable level and thus do not expect to change their clinical practice (Fig. 1c). The remaining 94% have a prior belief that 6-months trastuzumab delivers appropriate levels of DFS and are waiting for the evidence to be strong enough to be able to change their clinical practice.

### Perceptions of the relative efficacy of 6 and 12 months of trastuzumab in various subsets

**Fig. 2:**
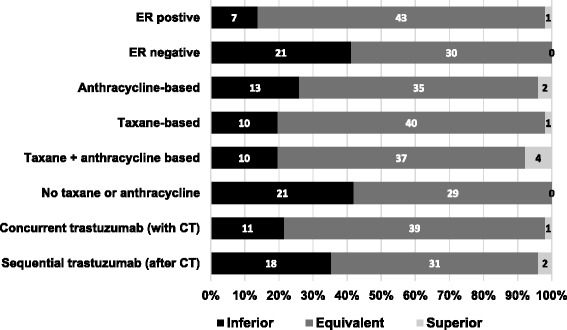
Opinions of the relative efficacy of 6 compared to 12 months of trastuzumab in various subsets

Eight subsets of breast cancer were listed and respondents were asked to indicate for each one whether they thought that, in terms of disease-free-survival, 6 months trastuzumab was an inferior treatment, an equivalent treatment or a superior treatment when compared to 12 months trastuzumab. The most common opinion expressed was of equivalent efficacy between 6 and 12 months of trastuzumab (Fig. 2), with between 58% and 84% of responders choosing this option across the different subsets. The subsets that gained the highest ratings for inferior efficacy with 6-months were the no anthracycline or taxane group (42%, 21/50), the ER negative group (41%, 21/51) and the sequential trastuzumab group (35%, 18/51). Four responders (8% of 51) rated 6-months of trastuzumab superior in efficacy to 12-months in the taxane and anthracycline sub-group.

Additional subsets proposed by responders with perceived equivalent efficacy between 6 and 12 months trastuzumab were lower grade (*n* = 1), small low grade (n = 1) and T1 N0 ER + ve HER2 + ve (n = 1). Additional sub-groups proposed by responders in which 6-months trastuzumab was perceived to have inferior efficacy to 12-months were (heavily) node positives (*n* = 4 respondents), patients in the neo-adjuvant setting (n = 1), patients receiving no chemotherapy (n = 1), higher grade (n = 1) and large high grade tumours (n = 1). One respondent perceived superior efficacy with 6-months trastuzumab in small node negatives.

### Perceived rates of cardiotoxicity

**Fig. 3:**
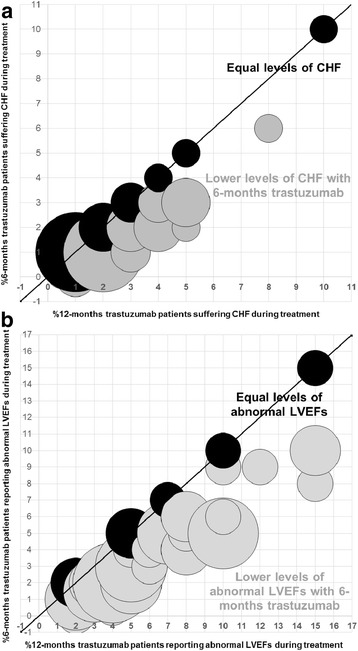
Perceived rates of patients reporting cardiotoxicity during treatment. **a** CHF: Clinically relevant congestive heart failure. **b** Abnormal LVEF: LVEF< 50% or an ECHO/MUGA classed as ‘abnormal’ by a cardiologist

When asked about their opinions on the percentage of patients who would suffer clinically relevant congestive heart failure (CHF) whilst receiving 12-months trastuzumab, responses ranged from 0.5% to 10% (median (inter-quartile-range (IQR)) 2% (1–4%)) (Fig. 3a). Opinions of such figures for patients receiving 6-months trastuzumab ranged from 0% to 10% (median (IQR) 1% (1–2%)). A third of responders (16/50 (32%)) perceived exactly equal rates of CHF across the two treatment durations. The remaining 34 responders perceived a reduction with 6-months trastuzumab, from 25% fewer to 100% fewer patients (i.e. no patients suffering CHF with 6-months trastuzumab).

Respondents’ perceptions of the percentage of patients reporting abnormal LVEF results during treatment were slightly higher. Responses for 12-months trastuzumab patients ranged from 2% to 15% (median (IQR) 5% (4–8%)) and, for patients receiving 6-months trastuzumab, ranging from 0% to 15% (median (IQR) 3.5% (2–5%)) (Fig. 3b). Only 14% of responders (7/50) perceived equal rates of abnormal LVEF reporting. The remaining 43 responders perceive a reduction with 6-months trastuzumab, from 10% fewer to 100% fewer (i.e. no patients reporting abnormal LVEF results with 6-months trastuzumab).

### Acceptable trade-offs of DFS and cardiotoxicity

**Fig. 4:**
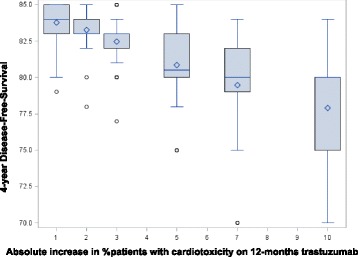
Acceptable 4-year DFS with 6-months trastuzumab to avoid hypothetical increased levels of cardiotoxicity

Respondents were asked to assume that 4-year DFS with 12-months trastuzumab was 85%. They were then presented with various hypothetical absolute increases in cardiotoxicity with 12-months trastuzumab when compared to 6-months and asked, for each proposed increased level, what 4-year DFS with 6-months trastuzumab they would find acceptable to achieve the lower level of cardiotoxicity. As the rates of patients reporting cardiotoxicity increased with 12-months trastuzumab, from 1%, 2%, 3%, 5%, 7% and 10% more, respondents reported median (IQR) acceptable levels of 4-year DFS with 6-months trastuzumab dropping to 84% (83–85), 83% (83–84), 83% (82–83), 80.5% (80–83), 80% (79–82) and to 80% (75–80) respectively (Fig. 4).

**Fig. 5:**
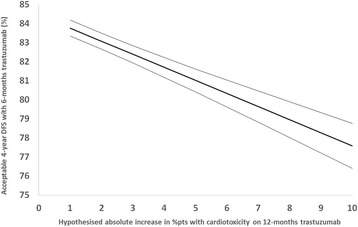
Random Effects modelling predicted lines and 95% CIs

As the hypothetical gap between the levels of patients suffering cardiotoxicity with 12 and 6-months trastuzumab widened, respondents accepted significantly lower levels of 4-year DFS with 6-months trastuzumab (Fig. 5, *p* < 0.0001).

### Additional comments

In the final free text section of the questionnaire, where respondents could write about their views on any aspect of the comparison between different durations of trastuzumab, comments were made on 4-year DFS (*‘I do not want to accept DFS <80%; hence I have not responded to x +7% and x+10% (above)’.* Comments also focused on cardiotoxicity (‘*Many believe 6 monthly Trastuzumab is (sufficient with) low cardiotoxicity’* and *‘I am of the opinion that most of the cardiotoxicity we see is reversible and not permanent’).* Respondents stressed how important the trade-off is between cardiotoxicity and DFS, and highlighted that it was a shared decision making process (*‘I have put my views but shared decision making requires the patient's views. Some will rate fear of recurrence very highly and not be so concerned about the risk of cardiac toxicity. Some will take the reverse view’).* One respondent emphasised their view that to change clinical practice the results would have to be near to equivalent (‘*the absolute benefit of Herceptin needs to be near equivalent with 6 months regimen compared with the 12 months for it to be acceptable to patients and clinicians’).*

## Discussion

Sixty percent of the responders to our questionnaire believe that 6-months trastuzumab treatment will afford exactly the same 4-year DFS as that expected with 12-months trastuzumab treatment (85% 4-year DFS). Almost all (94% of responders) expect to change their clinical practice to 6-months trastuzumab if 4-year DFS levels with 6-months trastuzumab are shown to be what they are expecting. The common consensus is that, across all the subsets of breast cancer, and types and sequence of chemotherapy, 6 and 12 months trastuzumab have equivalent efficacy. A minority of respondents believed that there might be some groups who would potentially still require 12 months trastuzumab. These included patients in the non-anthracycline or non-taxane groups, the group who received sequential trastuzumab after chemotherapy, and ER negative patients. The consensus prior belief therefore (including the minority respondents) is that if both anthracyclines and taxanes are given as chemotherapy, and concomitant trastuzumab is used (with the taxane component) that 6 months is equivalent / acceptably non-inferior to 12 months. In 2017, in the UK the majority of patients receiving adjuvant trastuzumab in this setting do already receive concomitant treatment, and anthracycline / taxane chemotherapy.

Since the trastuzumab duration questionnaire was circulated in July 2016 the results of a cardiology analysis has been published on 2500 Persephone patients [16] showing reported rates of clinical cardiac dysfunction as 12% and 9% for 12-month and 6-month trastuzumab patients respectively. Similarly, 12% and 9% of patients respectively reported low LVEFs. Responders to this questionnaire generally believed that cardiotoxicity rates would be lower than those we have reported, some even believing there would be no cardiotoxicity at all with 6-months trastuzumab. Now that these higher rates of cardiotoxicity have been shown, with both 6 and 12-months trastuzumab, there will be heightened interest in the DFS results of the Persephone trial (due mid-2018) from the perspective of balancing efficacy and cardiotoxicity.

The definitions of significant cardiotoxicity and even reduction in LVEF have varied considerably in the different trial reports. This could be one reason why responders prior beliefs of the rates of cardiotoxicity were lower than those actually reported from the Persephone trial. In the trastuzumab duration questionnaire, clinical cardiac dysfunction was defined as any symptoms or signs of congestive heart failure or the patient receiving any new or altered medication for cardiac disease. An abnormal LVEF was defined as LVEF < 50% or an ECHO/MUGA classed as ‘abnormal’ by a cardiologist. The HERA trial reported significant LVEF drops, defined as a decline in LVEF of > = 10% from baseline to < 50% [12]. In Persephone, such significant LVEF drops were reported by 9% of 12-month patients and 7% of 6-month patients [16]. Persephone also reported the percentage of patients with an LVEF< 50% after a baseline of > = 59% (6% of 12-month patients and 5% of 6-month patients [16]. Regardless of the definition used, in general the responders to our questionnaire underestimated the proportion of patients suffering cardiotoxicity with the two durations.

The trade-offs between cardiotoxicity and DFS are considerable. With proven absolute differences of 3% between cardiotoxicity levels with 12 and 6-months trastuzumab (both in terms of rates of patients reporting clinical cardiac dysfunction and in terms of patients reporting low LVEFs) [16] 83% 4-year DFS with 6-months trastuzumab is seen to be deemed acceptable by responders to this questionnaire (Fig. 4). This constitutes an absolute drop of 2% from the expected 85% 4-year DFS with 12-months trastuzumab. This is important information on prior clinical beliefs to help define endpoints in a planned meta-analysis of trastuzumab duration trials.

All endeavours to maximise the response rate in this study were undertaken, including multiple circulations of the survey to sites and multiple email reminders. However, it must be acknowledged that the small sample size within this study (51 respondents) remains a limiting factor in terms of the generalisability of the findings. In addition, the survey only included sites participating in the Persephone trial and this could also be viewed as a limitation since clinicians recruiting patients to the trial would continue to be in ‘clinical equipoise’ with regards to the six versus twelve month duration question. However, this survey was undertaken under the ethical approval obtained for the Persephone trial and thus the set of 152 recruiting sites defined the pool of participants available to us. Results from this survey were presented at the NCRI Cancer conference 2017 [17].

## Conclusion

There was an expectation from 94% (46/49) of responders that they would change their clinical practice to 6-months trastuzumab if their prior beliefs with regards to 4 years DFS were confirmed by the results of the Persephone trial. The most commonly held prior belief was that the lowest acceptable 4-year DFS was 83% with 6-months trastuzumab compared to 85% 4-year DFS for 12 months. Significantly lower levels of 4-year DFS with 6-months trastuzumab were deemed acceptable with hypothesised increases in cardiotoxicity. An individual patient meta-analysis of the duration trials would give greater precision to estimates of the differences in efficacy and toxicity, and adequate statistical power to establish a 2% level of non-inferiority for 6-months adjuvant trastuzumab.

## Additional file

Additional file 1: Trastuzumab Duration questionnaire. The questionnaire that was completed by the oncologists. (PDF 476 kb)

## Abbreviations

CHF: Congestive heart failure
CI: Confidence interval
DFS: Disease-free survival
IQR: Inter-quartile range
LVEF: Left ventricular ejection fraction
OS: Overall survival
