# Supplementary material for: Adjuvant trastuzumab duration trials in HER2 positive breast cancer – what results would be practice-changing? Persephone investigator questionnaire prior to primary endpoint results
Source: BMC Cancer. 2018 Apr 5;18:391. doi: 10.1186/s12885-018-4307-8 (PMC5887251; doi:10.1186/s12885-018-4307-8)
Supplement: Supplementary file 1 — Trastuzumab Duration questionnaire. The questionnaire that was completed by the oncologists. (PDF 476 kb) [file 12885_2018_4307_MOESM1_ESM.pdf]

Trastuzumab duration questionnaire

Name: ____

Hospital: ____

This questionnaire is designed to survey clinician's opinions on trastuzumab treatment in the HER2 positive early breast cancer setting, and more specifically the comparison between 12 months and 6 months duration of trastuzumab treatment. Its aim is to capture what clinicians consider both the effectiveness and toxicity profile of the two treatment durations.

Disease-Free Survival at 4 years

The PERSEPHONE trial assumed that patients receiving 12 months of trastuzumab would have a 4-year DFS of 85%. If this is proved accurate,

(a) … what 4-year DFS rate would you expect patients receiving 6 months trastuzumab to have?

(Please tick one box in column (a) to answer this part of the question)

(b) … what is the lowest 4-year DFS rate for 6 months trastuzumab patients that you would be comfortable with in order to change your practice to prescribing 6 months instead of the current standard of 12 months trastuzumab?

(Please tick one box in column (b) to answer this part of the question)

4-year DFS rate for 6 months trastuzumab patients	(a)	(b)
85%		
84%		
83%		
82%		
81%		
80%		
79%		
78%		
77%		
76%		
75%		
Other		

If 'Other' ticked above, please specify (a)______________________

(b)______________________

Disease-Free Survival in different subsets of patients

At the present time, in your opinion, in terms of disease-free-survival, is 6 months trastuzumab an inferior treatment, an equivalent treatment or a superior treatment when compared to 12 months trastuzumab in the following subsets of patients?

(Please tick one box in each row)

	Inferior	Equivalent	Superior
ER Positive patients	☐	☐	☐
ER Negative patients	☐	☐	☐
Patients receiving anthracycline-based CT (no taxane)	☐	☐	☐
Patients receiving taxane-based CT (no anthracyclines)	☐	☐	☐
Patients receiving taxane + anthracycline CT	☐	☐	☐
Patients receiving CT with no taxane or anthracycline	☐	☐	☐
Patients receiving concurrent trastuzumab and CT	☐	☐	☐
Patients receiving sequential trastuzumab (i.e. after all CT)	☐	☐	☐

Are there alternative/additional subsets of patients for whom you consider 6 months trastuzumab treatment to be either an inferior treatment, an equivalent treatment or a superior treatment, in terms of disease-free-survival, when compared to 12 months trastuzumab?
If so, please specify below.

(Please tick one box in each row)

	Inferior	Equivalent	Superior
________________	☐	☐	☐
________________	☐	☐	☐
________________	☐	☐	☐
________________	☐	☐	☐

Cardiotoxicity

In your opinion, what % of patients would, during their trastuzumab treatment, …	12 months trastuzumab	6 months trastuzumab
… suffer clinically relevant congestive heart failure† (CHF)?	_______%	_______%
… report an LVEF <50% or an ECHO/MUGA classed as 'abnormal' by a cardiologist?	_______%	_______%

† Clinically relevant CHF = symptoms of cardiac disease, or signs of congestive heart failure or receive medication for cardiac disease

<u>Trade-off of Disease-Free Survival and Cardiotoxicity</u>

We're now going to consider what decrease in DFS might be deemed as acceptable to avoid various ***hypothetical increased levels*** of cardiotoxicity (defined as LVEF <50% <u>or</u> an ECHO/MUGA classed as 'abnormal' by a cardiologist).

<u>Assuming 12 month trastuzumab gives a 4-year DFS of 85%</u>, how low a 4-year DFS would you accept as a trade-off to avoid the following ***hypothetical <u>absolute</u> increases*** in cardiotoxicity rates?

12 months trastuzumab	6 months trastuzumab
**4-year DFS = 85%**	**4-year DFS = ____%**
**x+1% of patients with cardiotoxicity**	**x% of patients with cardiotoxicity**

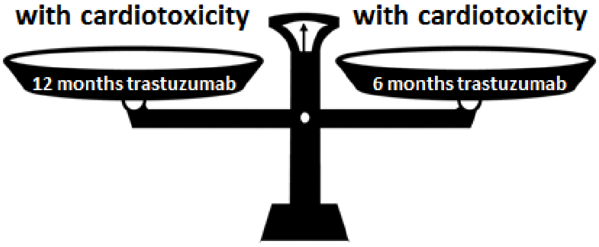

12 months trastuzumab	6 months trastuzumab
**4-year DFS = 85%**	**4-year DFS = ____%**
**x+2% of patients with cardiotoxicity**	**x% of patients with cardiotoxicity**

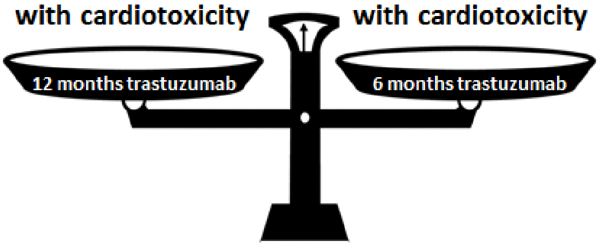

12 months trastuzumab	6 months trastuzumab
**4-year DFS = 85%**	**4-year DFS = ____%**
**x+3% of patients with cardiotoxicity**	**x% of patients with cardiotoxicity**

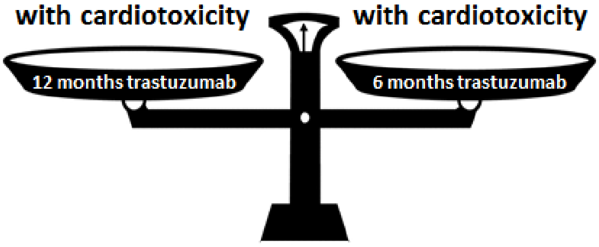

12 months trastuzumab	6 months trastuzumab
**4-year DFS = 85%**	**4-year DFS = ____%**
**x+5% of patients with cardiotoxicity**	**x% of patients with cardiotoxicity**

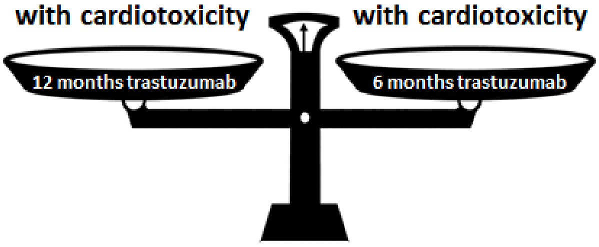

12 months trastuzumab	6 months trastuzumab
**4-year DFS = 85%**	**4-year DFS = ____%**
**x+7% of patients with cardiotoxicity**	**x% of patients with cardiotoxicity**

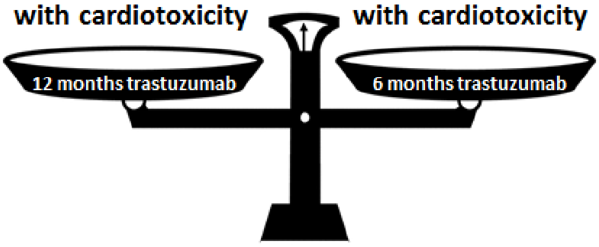

12 months trastuzumab	6 months trastuzumab
**4-year DFS = 85%**	**4-year DFS = ____%**
**x+10% of patients with cardiotoxicity**	**x% of patients with cardiotoxicity**

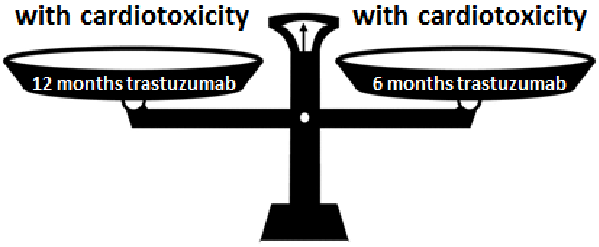

Please use the section below should you wish to add any comments

Thank you very much for taking time to consider the questions and complete this questionnaire.

Please return this questionnaire to Shrushma Loi, PERSEPHONE Trial Co-ordinator.

Via post: PERSEPHONE Trial
Warwick Clinical Trials Unit
Warwick Medical School
University of Warwick
Gibbet Hill
Coventry
CV4 7AL

OR

Via e-mail: Persephone@warwick.ac.uk

OR

Via fax: + 44 (0) 2476 151 586
